# Impacts of exposure to humidex on cardiovascular mortality: a multi-city study in Southwest China

**DOI:** 10.1186/s12889-023-16818-x

**Published:** 2023-10-04

**Authors:** Yang Li, Yizhang Xia, Hongbin Zhu, Chunli Shi, Xianyan Jiang, Shijuan Ruan, Yue Wen, Xufang Gao, Wei Huang, Mingjiang Li, Rong Xue, Jianyu Chen, Li Zhang

**Affiliations:** 1 Sichuan Provincial Center for Disease Control and Prevention, No.6, Zhongxue Road, Wuhou District, Chengdu, 610041 China; 2 School of Public Health, Chengdu Medical College, No.783, Xindu Road, Xindu District, Chengdu, 610500 China; 3 Chengdu Center for Disease Control and Prevention, No.6, Longxiang Road, Wuhou District, Chengdu, 610041 China; 4 Zigong Center for Disease Control and Prevention, No.826, Huichuan Road, Ziliujing District, Zigong, 643000 China; 5 Panzhi hua Center for Disease Control and Prevention, No.996, Jichang Road, Dong District, Panzhi hua, 617067 China; 6 Guangyuan Center for Disease Control and Prevention, No.996, Binhebei Road,Lizhou District, Guangyuan, 628017 China

**Keywords:** Comprehensive Index, Humidex, Cardiovascular disease, Temperature, Humidity

## Abstract

**Background:**

Many studies have reported the association between ambient temperature and mortality from cardiovascular disease (CVD). However, the health effects of humidity are still unclear, much less the combined effects of temperature and humidity. In this study, we used humidex to quantify the effect of temperature and humidity combined on CVD mortality.

**Methods:**

Daily meteorological, air pollution, and CVD mortality data were collected in four cities in southwest China. We used a distributed lag non-linear model (DLNM) in the first stage to assess the exposure–response association between humidex and city-specific CVD mortality. A multivariate meta-analysis was conducted in the second stage to pool these effects at the overall level. To evaluate the mortality burden of high and low humidex, we determined the attributable fraction (AF). According to the abovementioned processes, stratified analyses were conducted based on various demographic factors.

**Results:**

Humidex and the CVD exposure–response curve showed an inverted “J” shape, the minimum mortality humidex (MMH) was 31.7 (77th percentile), and the cumulative relative risk (CRR) was 2.27 (95% confidence interval [CI], 1.76–2.91). At extremely high and low humidex, CRRs were 1.19 (95% CI, 0.98–1.44) and 2.52 (95% CI, 1.88–3.38), respectively. The burden of CVD mortality attributed to non-optimal humidex was 21.59% (95% empirical CI [eCI], 18.12–24.59%), most of which was due to low humidex, with an AF of 20.16% (95% eCI, 16.72–23.23%).

**Conclusions:**

Low humidex could significantly increase the risk of CVD mortality, and vulnerability to humidex differed across populations with different demographic characteristics. The elderly (> 64 years old), unmarried people, and those with a limited level of education (1–9 years) were especially susceptible to low humidex. Therefore, humidex is appropriate as a predictor in a CVD early-warning system.

**Supplementary Information:**

The online version contains supplementary material available at 10.1186/s12889-023-16818-x.

## Background

Cardiovascular disease (CVD) has remained the main cause of death globally for the past 20 years [1], with > 17 million fatalities estimated yearly, accounting for almost 30% of all deaths worldwide and an associated disease burden of > 182 million disability-adjusted life years (DALYs) [2]. Although CVD mortality is decreasing in some developed countries, there has been no such change in developing ones [3], and the prevalence of CVD is continually increasing in China [4]. According to the China National Center for Cardiovascular Diseases (NCCD) [5], approximately 330 million Chinese people are affected by heart disease, accounting for roughly two out of five deaths. The aging population and rising CVD prevalence present China with a significant public-health concern. Therefore, to effectively prevent and intervene CVD, a thorough awareness of cardiovascular (CV) risk factors is imperative.

Numerous recent studies have investigated the relationship between temperature and humidity on the one hand and between temperature and the incidence of CVD on the other [6–8]; they have found that non-optimal (cold and hot) air temperatures can negatively affect the CV system [9–13]. However, findings on the relationship between relative humidity and CVD are inconsistent [14, 15]. The majority of current studies analyze only the effects of relative humidity or temperature alone after controlling for confounding factors. However, some researchers [12, 16] have found that there might be potential interactions between temperature and humidity and that the combined effects of meteorological factors might alter exposure–response relationships based on a single factor. Furthermore, temperature and humidity appear to interact differently in different climatic regions: a study in Iran [17] showed the highest frequency of doctor’s visits due to CVD in areas with low temperatures and low humidity, whereas a Chinese study [12] found that high temperatures and low relative humidity increase excess mortality by 10.18%. In addition, and importantly, humidity varies significantly from region to region due to topography, altitude, and other physical factors [18]. As a result, humidity impacts how humans perceive temperature, even when the actual air temperatures are similar [19]. Therefore, the use of ambient temperature as a representative indicator of weather’s effects on health remains questionable.

In this study, we used the composite index humidex as an alternative temperature indicator. This allowed us to quantify the combined impact of temperature and humidity on CVD mortality and take the effects of exposure to mixed climates into consideration. Humidex, established by the Canadian Meteorological Service [20], is widely used to evaluate climate comfort or to warn about perceived temperature since it takes into account two essential and fundamental components—temperature and humidity—that affect the degree of weather comfort [21]. Humidex has also recently been used in several environmental epidemiological studies, including in studies on the incidence of hand, foot, and mouth disease (HFMD) [22], bacillary dysentery [23], and childhood asthma [24]. However, because knowledge on the effect of humidex on CVD mortality is scarce, and the combined health effects of temperature and humidity are unknown, further research into humidex’s effect on CVD patients is necessary. When studied on their own, many factors have been found to be significantly associated with higher CVD death rates, including fine particulate matter, ozone levels, barometric pressure, and precipitation [25–28]. To obtain more accurate estimates, air pollution (such as fine particulate matter and ozone) and climactic factors (such as air pressure) were included as covariates in the time series and Poisson regression models used to assess the relationship between humidex and CVD mortality.

Sichuan Province is one of the highest-humidity regions in China. Therefore, we selected this region for this study, the goal of which was to examine the effect of humidex on CVD mortality and identify vulnerable populations via stratified analysis. We anticipate that the findings will enhance our understanding of the health hazards associated with multiple climatic factors.

## Methods

### Study areas

**Fig. 1:**
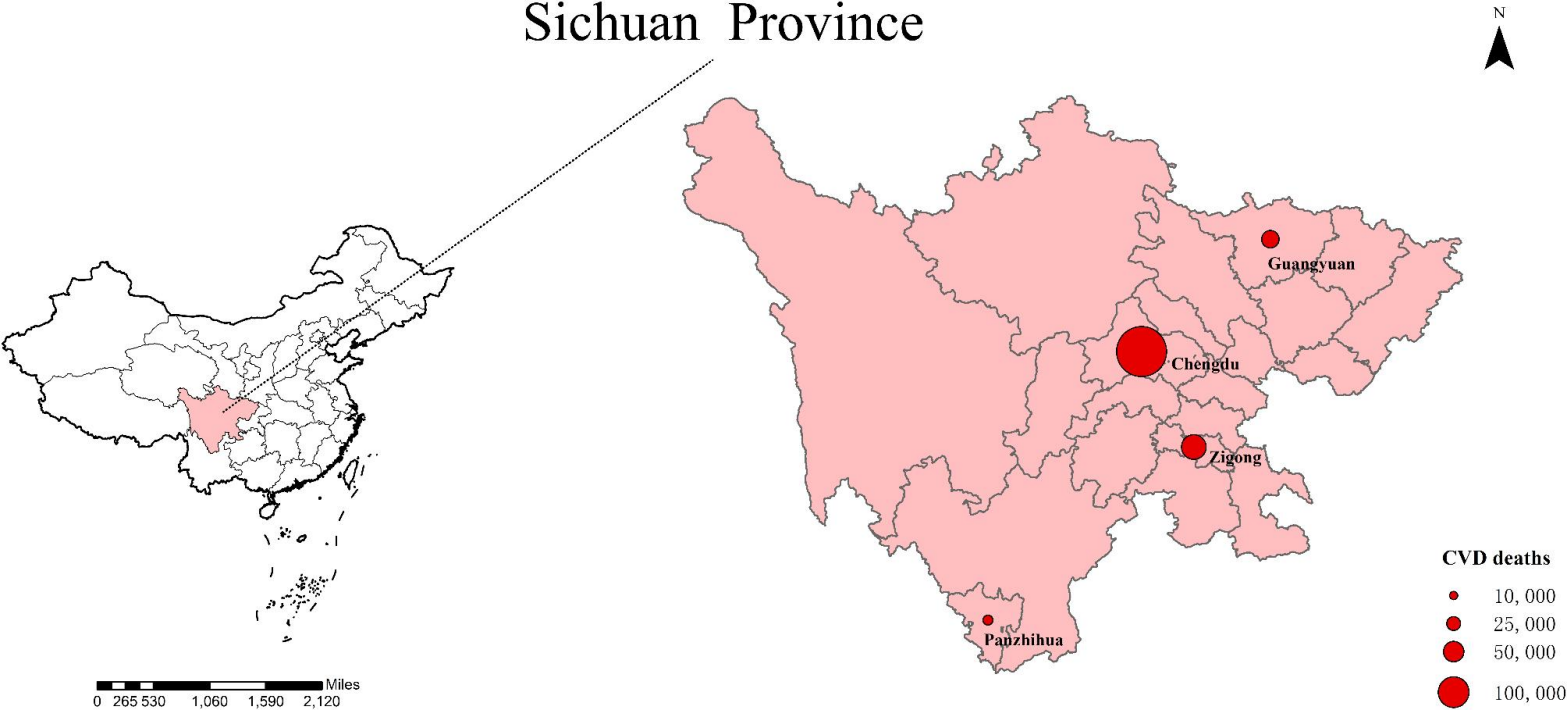
Locations of four cities in Sichuan Province and their city-specific cardiovascular disease (CVD) deaths. QGIS 3.30.1 software was used to create the map (QGIS Development Team, 2009; QGIS Geographic Information System)

Sichuan Province is located in the interior of southwest China, between 97°21’ and 108°33’E longitudes, and 26°03’ and 34°19’N latitudes. With elevations ranging from 359 to 7556 m, the terrain is high in the west and low in the east. Influenced by the topography and the alternation of different monsoonal circulations, the climate varies significantly from region to region in the province, and the climate type is mainly subtropical. In this study, we set up research sites in four cities of Sichuan Province: Chengdu (central), Zigong (southeastern), Guangyuan (northeastern), and Panzhihua (southwestern). Figure 1 shows their geographical distribution.

### Data resources

Data on daily mortality in the four cities were obtained from the China Cause of Death Reporting System (CDRS). Each record contains basic demographic information (age, gender, educational level, and marital status) and the date and primary cause of death. In the CDRS, CVD cases are classified according to the *International Classification of Diseases, 10th Revision* (ICD-10), I00–I99. Between January 1, 2016, and December 31, 2021, we collected 2192 records from Chengdu and Zigong, and 1461 records were collected from Guangyuan and Panzhihua between January 1, 2018, and January 1, 2021. To investigate the humidex-sensitive population, we stratified the data by gender (female and male); age (0–64 years and > 64 years); educational level (1–9 years and > 9 years); and marital status, which has two categories: married and other statuses (including never married, divorced, and widowed). Meteorological data were provided by the meteorological services of the cities studied, including daily average temperature (°C), daily average relative humidity (%), and average atmospheric pressure (hpa). We obtained environmental data from local environmental monitoring centers, including 24-h average fine-particulate matter (PM_2.5_; µg/m^3^) and daily 8-h maximum ozone concentrations (O_3–8 h_; µg/m^3^).

### Data quality control

Data reproducibility, integrity, and dependability were all subject to quality control (QC). We identified and eliminated duplicate data for critical factors such as address, date of birth, death date, and underlying cause of death. Age was calculated by the following formula: (death date − birthdate)/365.25. Meteorological data had no omissions. For both PM_2.5_ and O_3_, the overall percentage of missing data was < 1.4%, and missing values were interpolated using the average of three adjacent values.

### Statistical analysis

We used humidex, a temperature- and humidity-based composite index, as an exposure indicator in this study to better characterize human-perceived temperature (i.e., the actual temperature felt by a human being, rather than the objective air temperature). Humidex, proposed by Canadian meteorologists Richardson and Masterton [29], has been widely used in Canadian weather services [30]. In this study, the reference value of humidex corresponded to the minimum mortality humidex (MMH). Based on the cutoff values of the 5th and 95th percentiles of humidex and MMH, we divided humidex into four levels—extremely low humidex, moderately low humidex, moderately high humidex, and extremely high humidex—referring to previous studies [23, 31]. The formula was calculated as described in the following paragraphs.

1 $$Humidex={T}_{mean}+\frac{5}{9}\left(6.11\ast {10}^{(\frac{7.5{T}_{mean}}{237}+{T}_{mean})}\right)\ast \frac{RH}{100}-10$$

where *T* is the daily average temperature (°C) and *RH* is the daily relative humidity (%).

We conducted a classic two-stage analysis to evaluate the association between humidex and CVD mortality. In the first stage, we applied standard time series–adjusted Poisson regression models to quantify the exposure–response relationship between humidex and mortality in the given area. We included the following covariates to rule out the confounding effects of different seasons, holidays, air pollution, and other meteorological factors: (1) a natural cubic spline function of calendar time with 8 degrees of freedom (df) per year; (2) an indicator variable for the day of the week; and (3) a binary dummy variable for holidays; (4) Additionally, given that PM_2.5_ and O_3_ are the two most important air contaminants in Southwest China [32], and are associated with the occurrence of CVD [33], the matrix derived from the “cross-based” transformation of PM_2.5_ and O_3_ with 4 df was also included in the basic model. Spearman’s correlation coefficient (SCC) was used to examine the relationship between humidex and meteorological parameters, as well as between humidex and air pollutants. We excluded factors with an absolute value of *r* ≥ 0.7 to avoid co-linearity in the model [24]. Finally, the construction of the distributed lag non-linear model (DLNM) is described as follows:

Yt quasi-Poisson(E(Y))

2 $$\eqalign{{\rm{Log}}[E({{{Y}}_t})] & = {{a + cb(humide}}{{{x}}_{t,l}}{{) + cb(P}}{{{M}}_{2.5}}{{,df) + cb(}}{{{O}}_3}{{,df)}}\\& {{ + n}}{{{s}}_1}(pressure,df) + n{s_2}(time,df) + DOW + Holiday}$$

where *E(Yt)* represents the number of deaths from CVD at day *t*, *a* is the intercept, *cb(humidex*_*t,l*_*)* is the matrix derived by the “cross-based” transformation of daily humidex and the lag effect, *l* is maximum lag days, and *df* stands for degrees of freedom. Given the prolonged duration of the effect of cold weather, we defined the lag as 21 days in this study. “Cross-based” transformations were also performed for both PM_2.5_ and O_3_ due to potential confounders and the lag effects on health [34]. *cb(PM*_*2.5*_*)* and *cb(O*_*3*_*)* respectively denote the matrices of PM_2.5_ and O_3_ with 4 df. Furthermore, *ns* is the natural cubic spline, while *pressure* represents atmospheric pressure transformed by *ns* with 3 df. The long-term trend and seasonality were controlled by natural cubic spline with 8 df per year. *DOW* and *Holiday* stand for the day-of-the-week effect and the holiday effect. All model parameters were determined after considering the proposed Akaike information criterion (AIC) [35] and similar studies on CVD.

In the second stage of the analysis, we meta-merged the results of the first stage [36]; that is, we used a multivariate meta-analytical model to pool the overall cumulative exposure–response relationships after extracting the exposure coefficients for each city separately from the DLNM models. Given the different period lengths and population sizes in each study area, the best linear unbiased prediction (BLUP) was employed to obtain more-accurate cumulative exposure–response estimates. This approach enables areas with small daily mortality counts to borrow information from larger populations that share similar characteristics [36, 37]. Ultimately, we calculated the cumulative relative risk (CRR) of mortality from the CV system associated with humidex.

To demonstrate whether humidex or air temperature was a more appropriate indicator of the need for public-health intervention, we modeled mean daily temperature with the same parameters as for humidex. We also adjusted for the confounding effect of daily average relative humidity in the model with the *ns* function (3 df). Then, using a backward perspective within the DLNM framework, we calculated the attributable fraction (AF; *b* − *AF*_*x,t*_) and attributable number (AN; *b* − *AN*_*x,t*_) of humidex and temperature, taking into account risk at day *t* as the prior period’s (*t* − *l*_*0*_,…, *t* − *L*) cumulative exposure effects, with 95% empirical confidence intervals (eCIs) simulated by the Monte Carlo method [38]. Furthermore, we separately analyzed the attributable-death burden in subgroups divided by age, gender, marital status, and educational level using the two-stage model described above to identify populations potentially vulnerable to humidex. The formulae for calculation were as follows:

3 $${\rm b} - {AF_{x,t}} = 1 - \exp \left( { - \sum\limits_{l = {l_0}}^L {{\beta _{{x_{t - l},},l}}} } \right)$$

4 $${\rm{b}} - {AN_{x,t}} = b - A{F_{x,t}} \cdot {n_t}$$

where *b* − *AN*_*x,t*_, and *b* − *AF*_*x,t*_ are the correlation scores attributable to the number of cases with previous exposure to *x* and time *t*, *β*_*x,l*_ represents the sum of exposure contributions from *x*_*t–0*_,…, *x*_*t − l*_, and *n*_*t*_ is the number of cases at time *t*.

### Sensitivity analysis

To test the stability of the model, we performed several sensitivity analyses: (1) we changed the df of the time series in the model to 7, 8, and 9 to control for temporal trends; (2) we used different maximum lag days (7, 14, 21, and 28) to observe exposure–response relationships; (3) we adjusted df (3–5) for daily air pressure; and (4) we considered whether to include the air pollutants PM_2.5_ and O_3_ in the model; and (5) we plotted the exposure–response relationship between temperature and cumulative relative risk of CVD for different lag days.

For all data analyses, we used the dlnm and mgcv packages in R software (version 4.1.0; R Foundation for Statistical Computing, Vienna, Austria). A two-tailed *t*-test was used in all statistical analyses, and *P* < 0.05 was deemed statistically significant.

## Results

### Descriptive statistics

This study collected population, meteorological, and air pollution data for Chengdu and Zigong during 2016–2021 and for Guangyuan and Panzhihua during 2018–2021. We included 258,045 subjects aged 0–119 years, with 36 average daily deaths and 150 maximum daily deaths. The percentage of subjects aged 0–64 years was 15.04%, and that of subjects aged > 64 years was 84.96%. Percentages of males and females were 53.24% and 46.76%, respectively; 56.85% of the surveyed population was married, and 43.15% had other marital statuses. In terms of educational level, 91.29% of participants underwent ≤ 9 years, while 8.71% had > 9 years of schooling. During the research period, average temperature was 18.28 °C with humidex of 21.76, and average PM_2.5_ and O_3_ concentrations were 41.61 µg/m^3^ (3.79 µg/m^3^ – 300.75 µg/m^3^) and 85.43 µg/m^3^ (5.00 µg/m^3^ – 278.00 µg/m^3^), respectively. See Tables 1 and 2.

Daily mean temperature differs across the four cities, from 16.28 °C in Guangyuan to 21.58 °C in Panzhihua. Humidex ranges from 18.71 to 23.96 for all cities, with inter-city temperature variances more prominent in winter (Table S1).

**Table 1:** Descriptive statistics on meteorological indicators and air pollutants in four cities of Sichuan Province, China

Variables	Mean ± SD	Min	P(25)	P(50)	P(75)	Max
Humidex	(21.76 ± 11.08)	-4.85	12.40	21.89	31.19	47.33
Mean temperature (°C)	(18.28 ± 7.38)	-1.55	12.40	18.90	24.50	34.60
Mean relative humidity (%)	(72.37 ± 16.97)	11.80	64.00	76.00	84.93	100.00
Air pressure	(943.85 ± 37.28)	688.20	940.35	955.98	969.03	997.00
PM_2.5_ (µg/m^3^)	(41.61 ± 31.13)	3.79	21.17	32.63	51.02	300.75
O_3_(µg/m^3^)	(85.44 ± 39.45)	5.00	57.16	78.85	108.63	278.00

**Table 2:** Characteristics of all individuals accompanied by daily cardiovascular-disease mortality in four cities of Sichuan Province, China

Death Counts	Sum	Mean ± SD	Min	P(25)	P(50)	P(75)	Max
Total	258,045	(35.32 ± 30.50)	0	13.75	22	60	150
Gender							
Male	137,369	(18.81 ± 16.65)	0	7	12	31	85
Female	120,649	(16.51 ± 14.43)	0	6	11	27	73
Age (year)							
0–64	38,798	(5.31 ± 4.91)	0	2	4	8	26
> 64	219,247	(30.01 ± 26.21)	0	11	19	50	134
Educational attainment							
1–9 years	235,575	(32.24 ± 26.95)	0	13	21	53	136
> 9 years	22,470	(3.08 ± 4.05)	0	0	1	5	21
Marital status							
Married	146,704	(20.08 ± 17.23)	0	8	13	34	92
Others^a^	111,341	(15.24 ± 13.90)	0	5	10	25	73

^a^Others: other marital statuses include never married, divorced, and widowed

To avoid co-linearity in the model, we used SCC to assess the relationship between humidex and meteorological indicators or air pollutants. The results identified possible confounders: atmospheric pressure (*r*_*s*_ = − 0.34, *P* < 0.05), PM_2.5_ (*r*_*s*_ = − 0.46, *P* < 0.05), and O_3_ (*r*_*s*_ = 0.56, *P* < 0.05). Furthermore, because humidex was calculated using temperature and relative humidity, we removed these two factors from the regression model (see Table 3).

**Table 3:** Spearman’s correlation coefficients between cardiovascular-disease mortality and meteorological factors in four cities of Sichuan Province, China

Variables	CVD	Humidex	Temperature	Humidity	Air pressure	PM_2.5_
Humidex	-0.25^*^					
Temperature	-0.33^*^	0.97^*^				
Humidity	0.37^*^	0.05^*^	-0.14^*^			
Air Pressure	0.25^*^	-0.34^*^	-0.42^*^	0.30 ^*^		
PM_2.5_	0.32^*^	-0.46^*^	-0.44^*^	-0.02	0.29 ^*^	
O_3_	-0.05^*^	0.56^*^	0.63^*^	-0.45^*^	-0.33 ^*^	-0.09 ^*^

^*^P < 0.05

### Exposure–response relationship

**Fig. 2:**
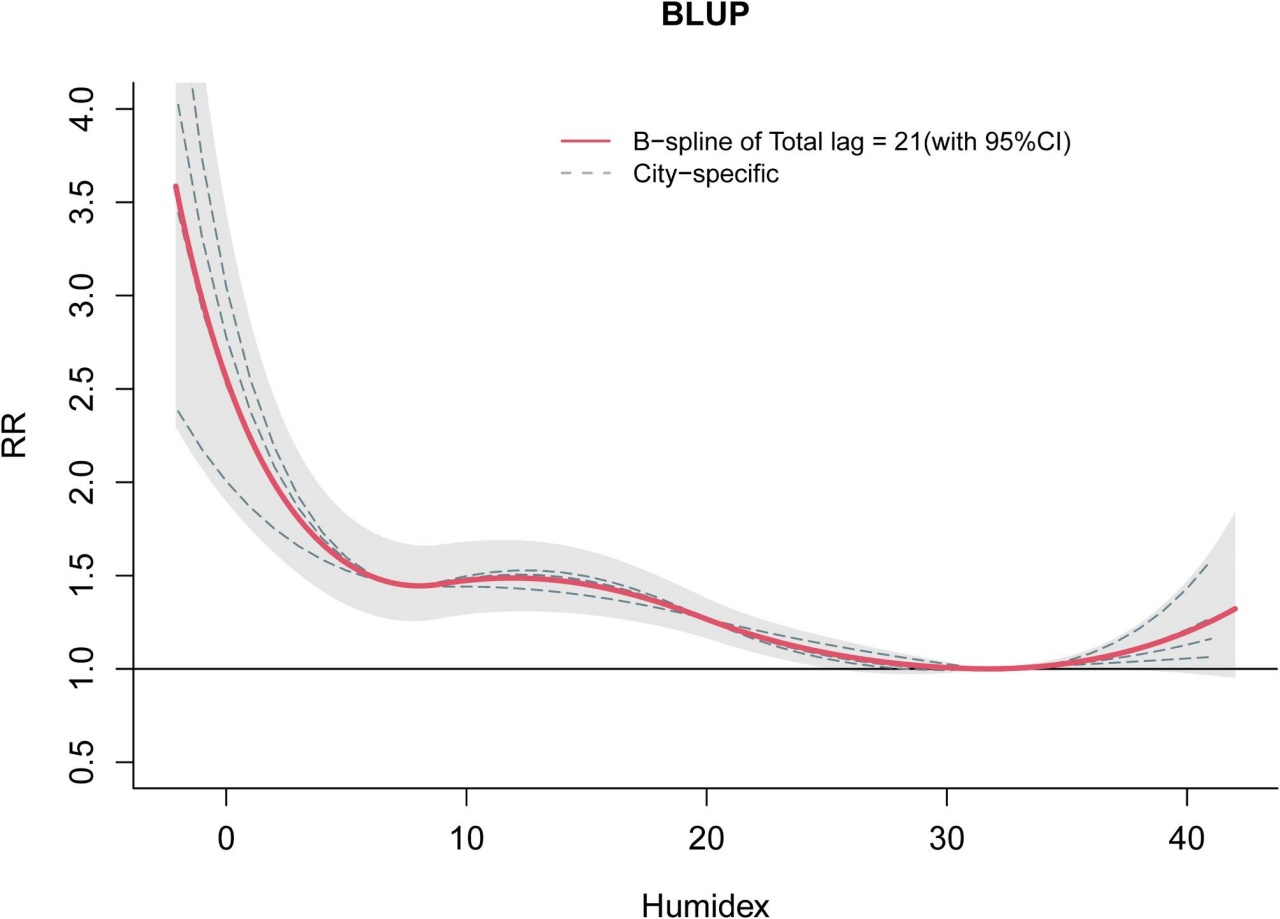
Exposure–response relationship between humidex and cumulative relative risk of cardiovascular-disease mortality in four cities of Sichuan Province, China. The dashed lines are the city-specific exposure–response curves, while the red line is the overall exposure–response curve after BLUP with lag of 0–21 days

Figure 2 illustrates BLUP estimates of the humidex–CVD mortality exposure–response relationship for all cities (lag, 0–21 days), with an approximate inverted “J” shape for humidex and mortality. Compared with MMH (31.7), the effects of both low and high humidex were significant. In particular, as shown by the non-linear, gradual change in its effect curve, low-humidex effect had a longer duration and lag time than high-humidex effect, whereas its high effect curve was transient and linear. We also observed that the slope of the curve sharply increased at both extremely high and low humidex levels, which suggested an increase in relative risk. Exposure–response curves for all cities were relatively similar, with low humidex having a stronger effect than high humidex (Fig S1).

**Fig. 3:**
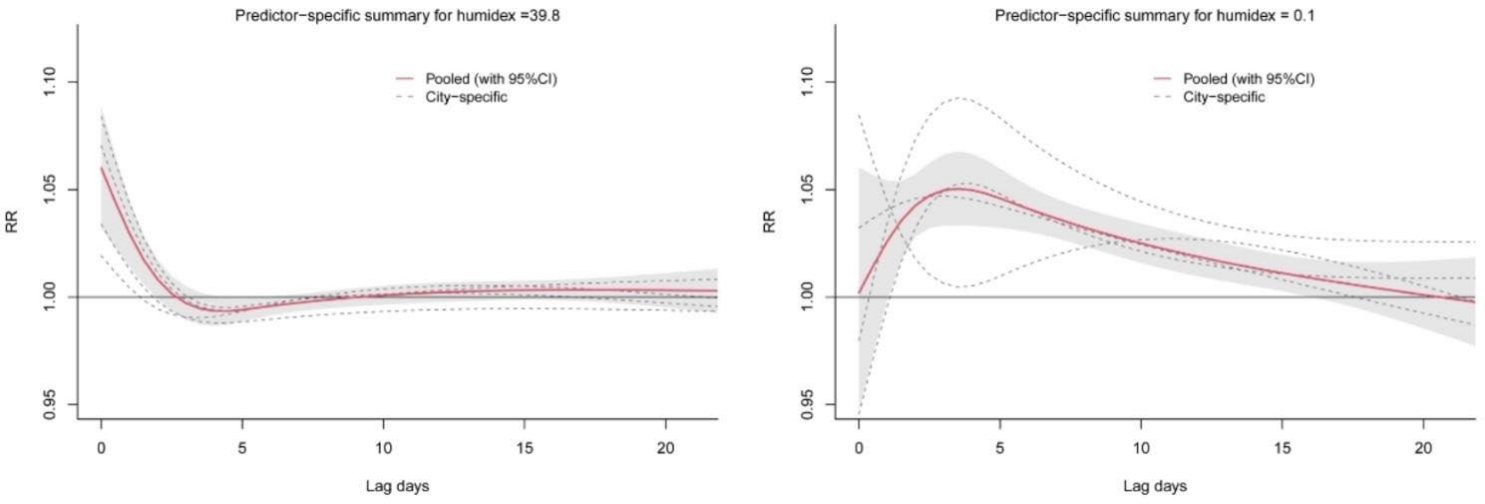
Lag–response relationship between extreme humidex and mortality in cardiovascular disease in four cities of Sichuan Province, China. The left and right panels respectively show the effects of extremely high humidex (39.8) and extremely low humidex (0.1) compared with estimated MMH (31.7)

In Fig. 3, the left panel shows the lag effect of extremely high humidex (39.8) on CVD mortality, which peaked on the day of exposure and lasted for 3 days (lag, 0–2 days), followed by a decrease in risk to a steady state that appeared to indicate the reduction in mortality. The right panel shows the lag effect of extremely low humidity (0.1), which reached its maximum on day 4 after exposure and persisted for nearly 3 weeks (lag, 0–21 days).

### Cumulative relative risks

Table 4 presents multiple meta-regression effects of extreme-humidex values on CVD mortality in different population subgroups. Overall MMH was 31.7 (77th percentile), and the CRR of CVD was 2.27 (95% CI, 1.76–2.91). Subjects who were male, aged 0–64 years, with ≤ 9 years of education, and of marital statuses other than married appeared to be more vulnerable to humidex and had a higher risk of CVD death; their CRRs were respectively 2.64 (95% CI, 1.37–5.08), 2.56 (95% CI, 1.76–3.74), 2.42 (95% CI, 1.84–3.17), and 2.90 (95% CI, 1.74–4.82). Extremely low humidex was also associated with an increased risk of CVD death (CRR, 2.52; 95% CI, 1.88–3.38), and sensitivity to extreme humidex varied among population subgroups. Risk of death was significant at extremely low and extremely high humidex in both females and subjects aged 0–64 years; for females, CRRs were 1.92 (95% CI, 1.48–2.49) and 1.65 (95% CI, 1.41–1.93), respectively, while for subjects aged 0–64 years, CRRs were 1.97 (95% CI, 1.25–3.11) and 1.27 (95% CI, 1.00–1.61), respectively. In terms of educational attainment, only subjects with 1–9 years of schooling were significantly susceptible to extreme low humidex, with a CRR of 2.66 (95% CI, 1.96–3.61). CRRs for each city are shown in Table S2.

**Table 4:** Meta-analysis of cumulative relative risks of cardiovascular-disease mortality due to extreme humidex in different subgroups of subjects in four cities of Sichuan Province, China

Group		MMH^a^	MMP^b^	Overall (95% CI)		Extremely low humidex (95% CI)		Extremely high humidex (95% CI)
Total		31.7	77th	2.27 (1.76–2.91)		2.52 (1.88–3.38)		1.19 (0.98–1.44)
Gender								
Male		37.5	89th	2.64 (1.37–5.08)		3.15 (1.53–6.50)		1.01 (0.90–1.13)
Female		31.3	76th	1.79 (1.40–2.29)		1.92 (1.48–2.49)		1.65 (1.41–1.93)
Age								
0–64		33.6	85th	1.80 (1.17–2.77)		1.97 (1.25–3.11)		1.27 (1.00–1.61)
> 64		29.9	89th	2.56 (1.76–3.74)		2.79 (1.85–4.22)		1.13 (0.86–1.48)
Educational attainment								
1–9 years		30.5	89th	2.42 (1.84–3.17)		2.66 (1.96–3.61)		1.15 (0.89–1.48)
> 9 years		33.7	85th	1.23 (0.71–2.15)		1.25 (0.70–2.25)		1.25 (0.92–1.70)
Marital status								
Married		34.0	85th	1.97 (1.51–2.58)		2.23 (1.64–3.05)		1.14 (1.00–1.29)
Others^c^		26.8	70th	2.90 (1.74–4.82)		3.13 (1.80–5.45)		1.41 (1.03–1.93)

^a^MMH: Reference values selected in our study. The humidex corresponding to the minimum mortality of cardiovascular disease; ^b^MMP: the corresponding percentiles of the minimum mortality humidex; ^c^Others: Other marital statuses include never married, divorced, and widowed

### Attributable fractions

**Fig. 4:**
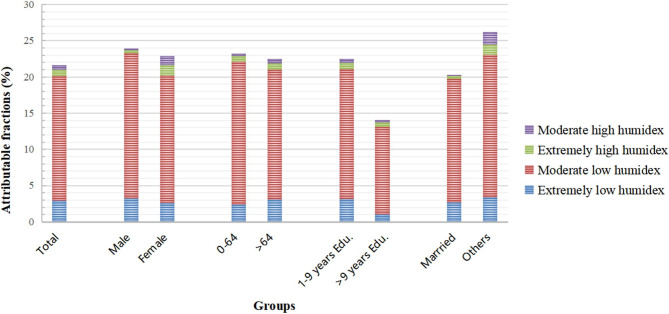
Attributable fractions of cardiovascular-disease mortality due to high and low humidex by gender, age, educational attainment, and marital status

Figure 4 visualizes the levels of AFs related to low and high humidex for different subgroups. City-attributable fractions are shown in Table S3. A total of 21.59% of CVD deaths were attributable to humidex. The results of both levels of humidex clearly indicated that low humidex caused the most CVD deaths. AFs of high and low humidex were 1.43% (95% eCI, 0.83–1.94%) and 20.16% (95% eCI, 16.72–23.23%), respectively. Further refinement of high or low humidex into moderate and extreme levels showed that the majority of the mortality burden was attributable to moderate humidex, while extreme humidex accounted for only a small proportion of that burden. In the total population, the greatest burden of death attributable to humidex was in those with 1–9 years of education, marital statuses other than married, aged 0–64 years, and male gender, with overall AFs of 22.52% (95% eCI, 17.46–26.29%), 20.31% (95% eCI, 15.56–24.55%), 23.22% (95% eCI, 12.75–30.85%), and 23.89% (95% eCI, 10.87–31.50%), respectively.

At the city level, high humidex posed the highest risk in Chengdu, at 1.74% (95% eCI, 0.90–2.47%), while posing the lowest risk in Guangyuan, at 0.64% (95% eCI, − 0.39 − 1.71%). However, Guangyuan differed from the other three cities in attribution components of humidex: CVD risk there was more attributable to moderate humidex, whereas the remaining cities were more affected by extreme humidex (Table S3).

### Sensitivity analyses

Sensitivity analysis showed that the relationship between humidex and CVD mortality and related trends were very similar when using different lag times, adjustments for time trends, different dfs of atmospheric pressure, and alternative controls for air pollutants in the model. This indicated that this model was reliable and stable (Fig S2).

The exposure–response curve for humidex followed a trend similar to that of the temperature for various lag days. This revealed that humidex is stable enough to be used as an alternative index of temperature in prediction. Notably, humidex had a higher absolute value than the temperature recorded by the meteorological station, as well as a greater overall CRR value than the temperature; the CRR values for humidex and temperature were 2.27 (95% CI, 1.76, 2.91) and 1.86 (95% CI, 1.34, 2.59), respectively (Fig S3). Attributable risk and attributable number associated with humidex were higher than those associated with temperature (humidex: *b* – *AF* = 21.59% [95% eCI, 18.12–24.59%], *b* – *AN* = 55,717; temperature: *b* – *AF* = 20.48% [95% eCI, 16.67–24.22%], *b* – *AN* = 52,857). This suggested that humidex seemed to be a better predictor of CVD mortality than temperature (Table S4).

## Discussion

To the best of our knowledge, this was the first study to use a combined indicator to associate the combined effects of ambient temperature and relative humidity on CVD mortality in the Sichuan Basin of China. The association was stronger than if we had used a single meteorological indicator, and therefore the combination was much more effective at indicating threats to health.

We discovered that both low and high humidex increased the risk of mortality after deviation from optimal humidex (MMH, 31.7): 21.59% of CVD deaths were attributable to humidex, of which low humidex accounted for 20.16%. This value exceeded that obtained in a previous study based on 272 Chinese cities, which found that temperature was responsible for 17.48% of CVD mortality [39]. Previous research has suggested that temperature and humidity might interact and that their effect on CV health differs by region [23, 24]. For example, Zeng et al. found that low temperature and high humidity increased the risk of CV mortality in coastal China [40], whereas Fang et al. observed that high temperature and low humidity had a more significant impact on CVD health occurrences in central China [16].

Although we do not fully understand the biological mechanisms by which temperature and humidity affect human health, existing evidence suggests that both have non-linear relationships to health and that their combined effect might greatly exceed the sum of their individual effects [12, 41]. According to an experimental study [42], thermal sensation increased by 0.63 scalar units at the same temperature when relative humidity was raised from 70 to 90%, which means that the human body’s relationships to cold and heat (sensation, acceptability, and comfort), as well as its physiological responses, change considerably as relative humidity increases. Physiological data show that in a hot environment, the body adjusts its core temperature mostly through perspiration [43, 44]. However, high humidity can inhibit the cooling process to some extent, particularly in a warm, humid climate, because heat loss through evaporation is reduced. When body temperature is too high, it may predispose the individual to autonomic nervous-system malfunction and cause CV problems [45, 46]. Cold exposure might enhance catecholamine levels and serum cholesterol, resulting in vasoconstriction and an increase in blood pressure (BP), predisposing the individual to angina or myocardial infarction [47]. Furthermore, cold, wet weather can cause airway oversaturation, influencing the generation of clotting factors, which can contribute to CVD [48].

We discovered that the effect of low humidex on CVD was stronger than that of high humidex, not only because the effect on mortality increased but also because the delayed effect of low humidex lasted longer. When humidex reached extreme lows (< 0.1), the relative risk of death increased significantly from the day of exposure and lasted for 21 days. In contrast, the effects of extremely high humidex were short-lived, only 3 days. Yang et al. [49] noted that patients had significantly higher BP and greater CVD mortality in winter. A study of 567 cities in 27 countries by Alahmad et al. [50] also found that exposure to extreme temperatures was related to increased risk of death from several common CVDs, with low temperatures presenting the most health concerns and with longer lags. These findings suggest that people with CVD should be monitored and treated more aggressively during the colder months.

The findings of our study indicated that the lowest relative-mortality risk corresponded to 77th percentile humidex, which was lower than all-cause mortality. This suggested that Sichuan Basin residents might have a greater likelihood of non-optimal humidex exposure, as well as a wide range of sensitivity to humidex. CRR at extremely low humidex was 2.52 (95% CI, 1.88–3.38), which was similar to the findings of a large multi-country study but slightly higher than that observed in some regions such as Greece [51], Brazil [52], and Beijing [53]. There are two potential explanations for this discrepancy. The first is that it might be due to variations in study region and population characteristics. One study proved that latitude and longitude strongly correlate with risk of CV mortality [54], and another showed that population temperature adaptations might also lead to differences [51]. The second possible explanation is different definitions of extreme values. Previous studies used the 1st percentile of temperature to define extremely cold weather. Using more-extreme percentiles results in less data being included in the analysis, potentially weakening the relationship between extreme cold and CVD.

AF, the proportion of cases that can be attributed to a risk or exposure factor and that could be reduced through public-health interventions, is significant for prioritizing disease management and potential consequences. The AF of CVDs was 21.59% in our study, and it was much broader at low than at high humidex, respectively 20.16% and 1.43%. This finding was supported by previous research and added to the evidence that cold weather increases health risks more than hot weather [38, 55]. As shown in Fig. 4, proportions of AFs at different levels of humidex showed that the majority of AFs exhibited low humidex in all subgroups, while high humidex resulted in low CRRs and AFs. A recent epidemiological study obtained the same results [56]. These findings might be related to improvements in healthcare systems and housing conditions (e.g., air conditioning, better treatments for heat-related morbidity). Our findings provided epidemiological support for health hazards in various climatic environments, as well as for recommendations that health risk warnings be increased on moderately cold winter days.

Previous epidemiological evidence of vulnerable populations in extreme climates is inconsistent. We noted that males and people > 64 years old were more sensitive to very low humidex, while females and those 0–64 years old were more susceptible to extremely high humidex. Similar findings were obtained in studies from Japan [45], Spain [57], and Greece [51], while other studies have reported the opposite [39, 56]. Variations in populations’ physiological and behavioral adaptations to local climate conditions, as well as differences in health-related factors (e.g., government climate adaptation policy), could explain this regional heterogeneity [58]. Women’s higher body fat ratio, for example, might be one reason they are more resistant to cold climates [56]. The availability of central heating and governmental strategies to adapt to climate change might also affect the results. In general, the elderly are thought to be more vulnerable to climate extremes, which our findings supported. This is likely due to their lower thermoregulatory capacity, lower ability to adjust to changes in ambient temperature, and the inability to access medical advice or public-health services in a timely manner [55]. It is worth noting that AFs were higher in the age 0–64 group. This indicated a wide range of sensitivity to climate change in the young; therefore, if health alerts were broadcast to the general population, additional health gains might result.

Another finding in this study was that different marital statuses and educational levels were also associated with risk of death from CVD. We showed that CVD mortality was increased in people whose marital statuses were other than married. Many studies [59–61] have indicated that socioeconomic support and health-promoting activities in marriage have a positive influence on health, and this protective effect is especially obvious in Asian marriages that emphasize the stability of the institution and other family ties [62]. At the same time, we found that populations with fewer years of education were more vulnerable to climate change and had higher risk of CVD mortality. Meanwhile, we did not see significant evidence of high- or low-humidex effects in subjects with > 9 years of education. Although our results were consistent with those of a Spanish study [63], subjects with 0–9 years of education represented 91% of the total population we surveyed; those with > 9 years of education were only a small percentage of our cohort. The overrepresentation of the former in our results might have overly elevated our CRR values at extreme humidex and caused us to somewhat overestimate the negative health effects of limited education. However, the results are still informative.

Similar to previous studies [19, 22], we found that the value of humidex was higher than that of the temperature recorded by the meteorological station, and that the CRR and AF values outperformed temperature alone in prediction. These findings imply that humidex could have broader and more sensitive predictive properties for CVD death, thus providing us with new insights to quantifying the weather–CVD association.

Several limitations of this study should be noted. First, data on individual exposures were unavailable. The temperature and humidity data used in the humidex computation were collected from fixed-site outdoor monitors and reflected the total exposure level of residents in a given area. Although this method is widely used in environmental epidemiology studies, it does not account some adaptive behaviors, such as air conditioning use, outdoor-exposure reduction, or local preventive and intervention initiatives, which can lead to misclassification. Second, the Sichuan Basin is one of China’s highest-humidity regions, and while the cities included in this study did not show significant heterogeneity in the multiple-regression analysis results, caution is advised when extrapolating these findings to other regions, particularly those in different climate zones. More data from more regions are required to clarify the more-general combined effects of temperature and humidity. Third, we used humidex to reflect the effect of weather, but there might possibly be a superior indicator for characterizing the influence of climate on CVD. Finally, we used an ecological study design, which is vulnerable to ecological fallacies [64].

In conclusion, climate change presents new challenges to public health, and vulnerable populations should be given priority in CVD prevention and interventions. These include those who are male, > 64 years old, not married, and with low educational levels.

## Conclusions

This multi-city study examined the relationship between humidex and CVD mortality in southwest China. Our findings showed that humidex was correlated with CVD mortality in the Sichuan Basin, particularly low humidex, which explained the majority of CVD deaths. Subjects with marital statuses other than married were more sensitive to extreme humidex than the married population. Males and subjects > 64 years old were more vulnerable to extremely low humidex, while females and subjects aged 0–64 years were more susceptible to extremely high humidex. By identifying humidex-vulnerable populations, these findings support the inclusion of humidex in CVD risk warning systems and have significant implications for future public-health interventions to reduce weather-related health risks.

### Electronic supplementary material

Below is the link to the electronic supplementary material.

Supplementary Material 1

## Data Availability

The underlying data in this article will be shared upon reasonable request to the corresponding authors.

## Abbreviations

CV: Cardiovascular
CVD: Cardiovascular disease
PM_2.5_: Particulate matter ≤ 2.5 µm in aerodynamic diameter
RH: Relative humidity
MMH: Minimum mortality humidex
MMP: Minimum mortality percentile
DLNM: Distributed-lag non-linear model
SD: Standard deviation
CRR: Cumulative relative risk
CI: Confidence interval
eCI: Empirical confidence interval
AF: Attributable fraction
BLUP: Best linear unbiased prediction
