# Supplementary material for: Impacts of exposure to humidex on cardiovascular mortality: a multi-city study in Southwest China
Source: BMC Public Health. 2023 Oct 4;23:1916. doi: 10.1186/s12889-023-16818-x (PMC10548730; doi:10.1186/s12889-023-16818-x)
Supplement: Supplementary file 1 — Supplementary Material 1 [file 12889_2023_16818_MOESM1_ESM.docx]

**Additional file 1**

Table S1. Descriptive statistics of the Meteorological factors, air pollutants, and cardiovascular diseases daily deaths in four cities of Sichuan Province, China, (mean ± standard deviation).

Table S2. Cumulative relative risk attributable to extreme humidex in four cities of Sichuan Province, China.

Table S3. Attributable fractions of CVD mortality due to high and low humidex in four cities of Sichuan Province, China.

Table S4. Backward attributable risk fractions (b-AF) and numbers (b-AN) of humidex and mean temperature.

Fig. S1 Exposure–response associations between humidex and CVD mortality in four cities of Sichuan Province, China.

Fig. S2 Exposure–response curves with different lag times and a variety of degrees of freedom of time, atmospheric pressure, and air pollutants.

Fig. S3 Exposure–response curves with different lag days for extremely high and low temperatures.

Table S1. Descriptive statistics of the Meteorological factors, air pollutants, and cardiovascular diseases daily deaths in four cities of Sichuan Province, China, (mean ± standard deviation).

| City | Study period | Death Counts | Humidex  (range) | Mean temperature (°C)  (range) | Mean relative humidity (%)  (range) | PM2.5 (µg/m^3^)  (range) | O3-8h(µg/m^3^)  (range) |
| --- | --- | --- | --- | --- | --- | --- | --- |
| Chengdu | 2016-2021 | 170,381 | 20.52±11.40 (-4.85, 42.15) | 16.82±7.40 (-1.55, 30.45) | 79.70±9.44 (36.00, 99.00) | 47.43±33.05 (3.79, 256.52) | 94.16±47.17 (10.28, 278.00) |
| Zigong | 2016-2021 | 50,193 | 23.55±11.60 (-1.73, 47.33) | 18.86±7.39 (0.80, 34.60) | 78.65±12.05 (34.00, 100.00) | 53.96±37.37 (7.75, 300.75) | 82.74±38.55 (13.00, 250.63) |
| Guanguan | 2018-2021 | 27,162 | 18.74±11.46 (-4.66, 40.56) | 16.28±7.64 (-1.00, 29.70) | 70.07±14.62 (14.00, 99.00) | 26.50±17.18 (5.50, 132.00) | 72.94±31.60 (5.00, 193.00) |
| Panzhihua | 2018-2021 | 10,309 | 23.96±8.05 (2.64, 37.98) | 21.58±5.56 (4.80, 33.60) | 54.22±20.02 (11.80, 98.30) | 29.43±12.73 (7.29, 119.14) | 88.91±30.42 (15.29, 183.14) |

Table S2. Cumulative relative risk attributable to extreme humidex in four cities of Sichuan Province, China.

| City | MMH^a^ | MMP^b^ | Overall | Extremely low humidex | Extremely high humidex |
| --- | --- | --- | --- | --- | --- |
| Chengdu | 32.1 | 79th | 1.87 (1.45, 2.42) | 1.64 (1.37, 1.95) | 1.19 (1.07, 1.31) |
| Zigong | 34.9 | 79th | 2.11 (1.58, 2.80) | 1.44 (1.23, 1.68) | 1.28 (0.91, 1.81) |
| Guangyuan | 27.8 | 72th | 2.77 (1.90, 4.05) | 2.42 (1.76, 3.34) | 1.03 (0.95, 1.11) |
| Panzhihua | 31.4 | 75th | 2.42 (1.69, 3.47) | 1.51 (1.31, 1.73) | 1.03 (0.98, 1.08) |

^a^MMH: the humidex corresponding to the minimum risk of CVD mortality; ^b^MHP: corresponding percentiles of the minimum mortality humidex.

Table S3. Attributable fractions of CVD mortality due to high and low humidex in four cities of Sichuan Province, China.

| City | Overall | Low humidex | | |  | High humidex | | | |
| --- | --- | --- | --- | --- | --- | --- | --- | --- | --- |
|  |  | Low-overall | Moderate low | Extremely low |  | High-overall | Moderate high | | Extremely high |
| Chengdu | 21.68（17.16, 25.82) | 19.94（15.13, 24.63) | 17.20（12.8, 21.32) | 2.74（2.04, 3.33) |  | 1.74（0.90, 2.47) | | 0.72（0.34, 1.08) | 1.03（0.52, 1.47) |
| Zigong | 21.03（16.46, 24.76) | 20.05（15.02, 24.22) | 16.93（12.55, 20.80) | 3.12（2.40, 3.68) |  | 0.98（0.05, 1.79) | | 0.40（-0.01, 0.80) | 0.57（-0.01, 1.10) |
| Guangyuan | 22.47（18.31, 26.03) | 21.83（17.47, 25.63) | 17.92（14.09, 21.36) | 3.95（3.17, 4.56) |  | 0.64（-0.39, 1.71) | | 0.40（-0.27, 1.04) | 0.25（-0.55, 0.84) |
| Panzhihua | 20.63（16.46, 24.31) | 19.89（15.47, 23.87) | 16.99（13.15, 20.53) | 2.93（2.22, 3.49) |  | 0.74（-0.35, 1.70) | | 0.29（-0.14, 0.69) | 0.45（-0.45, 1.23) |

Table S4. Backward attributable risk fractions (b-AF) and numbers (b-AN) of humidex and mean temperature.

| Index |  | Overall |
| --- | --- | --- |
| Humidex | b-AF | 21.59% (18.12%, 24.59%) |
|  | b-AN | **55717** |
| Mean temperature | b-AF | 20.48% (16.67%, 24.22%) |
|  | b-AN | 52857 |

*AF: attributable risk fractions; AN: attributable risk numbers.


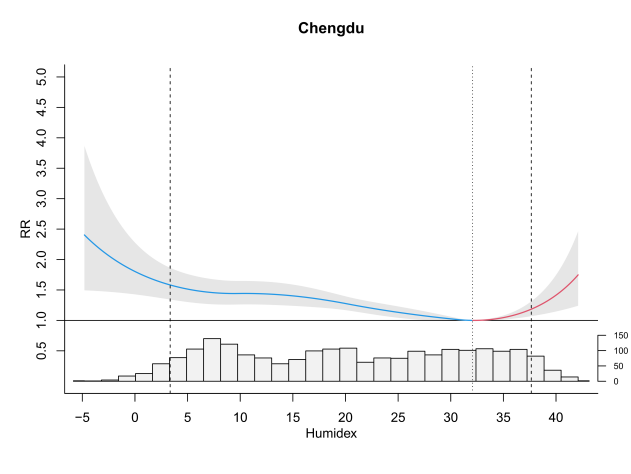

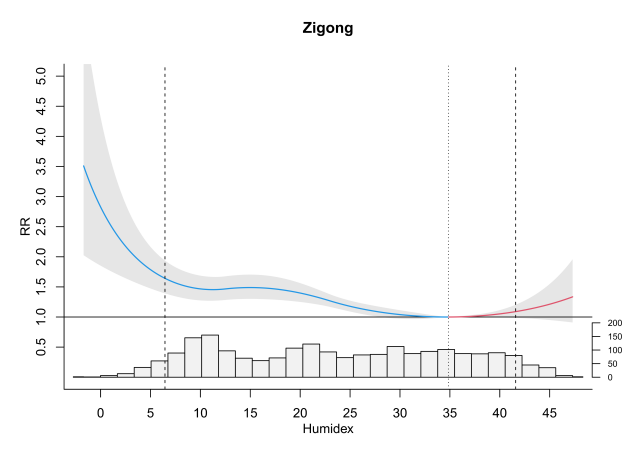


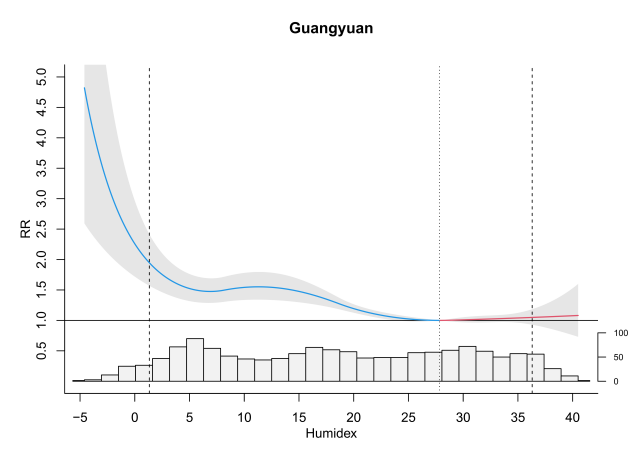

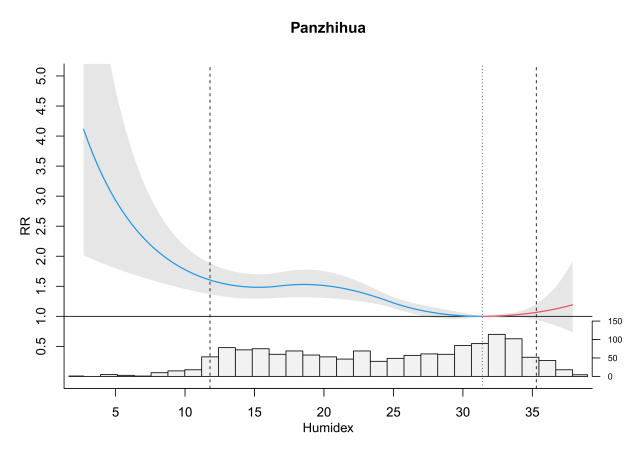


Fig. S1 Exposure-response associations between humidex and CVD mortality in four cities of Sichuan Province, China. The light grey dotted line is the humidex corresponding to the minimum CVD mortality, and the black dotted line is the 5th and 95th percentile, respectively. The blue and red lines represent the low and high humidex effects, respectively (with 95% CI, shaded grey). The bar graph shows the frequency distribution of humidex.


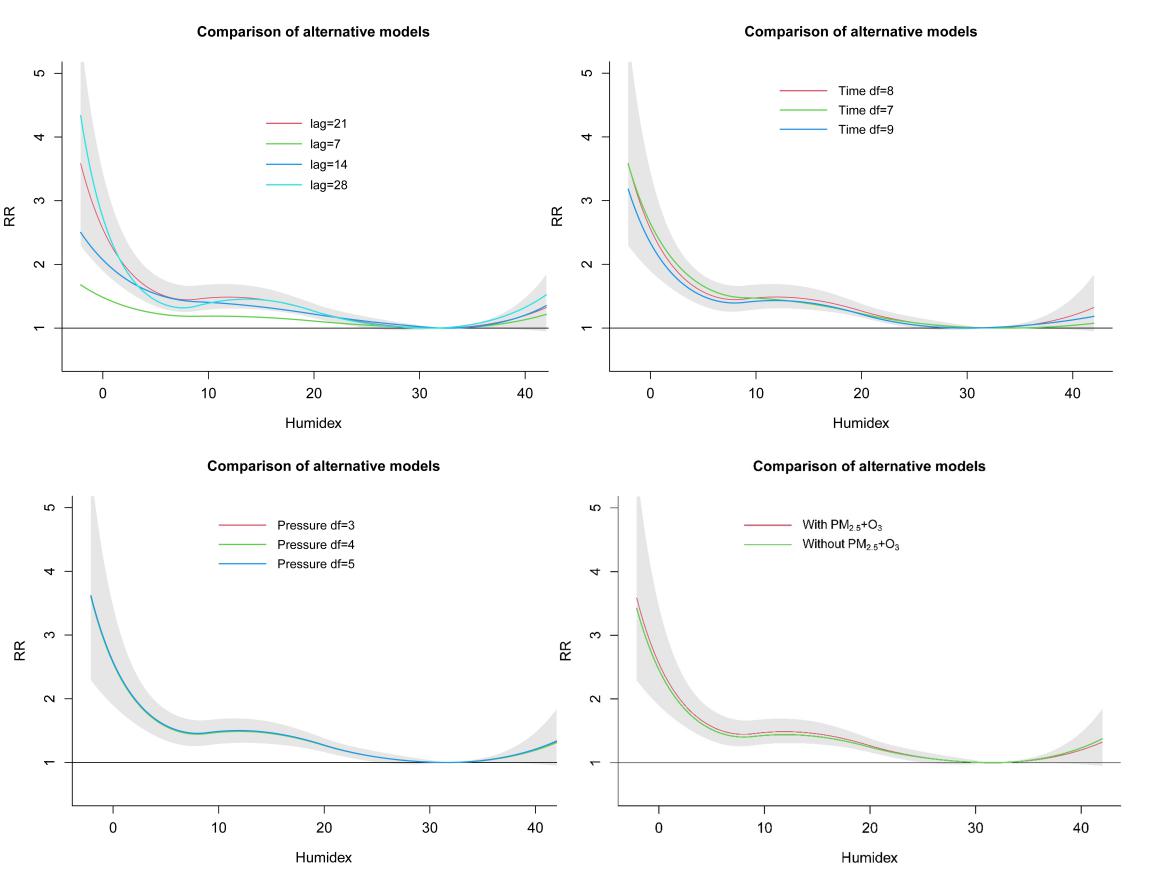


Fig. S2 Exposure–response curves with different lag days and a variety of degrees of freedom of time, atmospheric pressure, and air pollutants.


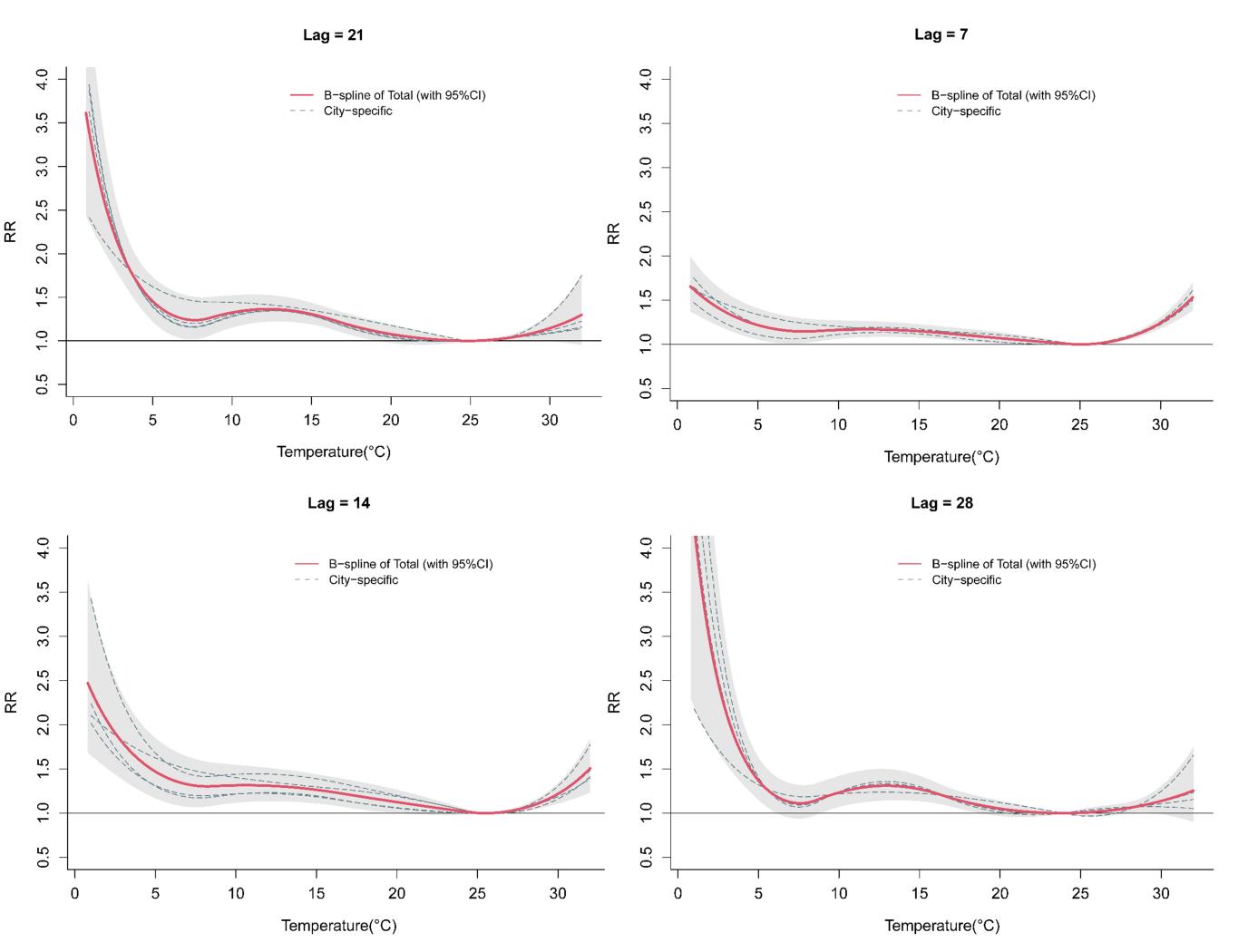


Fig. S3 Exposure–response curves with different lag days for extremely high and low temperatures.
